# Small incision discectomy for lumbar disc herniation in 98 patients with 5-year follow-up: A retrospective case series study

**DOI:** 10.1097/MD.0000000000015569

**Published:** 2019-05-13

**Authors:** Zhinan Ren, Zheng Li, Shugang Li, Derong Xu, Xin Chen

**Affiliations:** a Department of Orthopaedics, Peking Union Medical College Hospital, Chinese Academy of Medical Sciences and Peking Union Medical College, Dongcheng District, Beijing; b Department of Orthopaedics, The First Affiliated Hospital of Zhengzhou University, Zhengzhou, China.

**Keywords:** lumbar disc herniation, minimally invasive discectomy techniques, small incision discectomy

## Abstract

Optimal surgical technique to treat lumbar disc herniation (LDH) remains controversial. We described a small incision discectomy technique (SID), and to evaluate its safety and efficacy. A retrospective study involving 98 consecutive patients with LDH managed by SID was conducted. All patients were followed up for 5 years. Outcomes included visual analogue scale (VAS), Japanese Orthopedic Association (JOA), operative time, length of incision, blood loss, hospital stay, hospitalization costs, x-ray exposure, reoperation, and complications. The results were determined to be excellent, good, fair, or poor according to the MacNab classification. All patients completed the 5-year follow-up. Relative to preoperative scores, VAS and JOA were both significantly improved. As a whole, 93.8% (92/98) patients showed excellent or good results, 3.1% (3/98) fair, and 3.1% (92/98) poor. The operation time, length of incision, blood loss, and hospital stay were 50 ± 11.1 minutes, 2.2 ± 0.3 cm, 35 ± 3.5 mL, and 4.3 ± 0.2 days, respectively. Additionally, compared with previous literature reports, the hospitalization costs and x-ray exposure were apparently less. The reoperation and recurrence rate were 3.2% and 2.1%. No complications were observed. From these data we conclude that SID appears to be a safe, cost-effective technique for LDH, and has lower x-rays exposure time when compared with literature of percutaneous endoscopic lumbar discectomy (PELD).

## 1 Introduction

Symptomatic lumbar disc herniation (LDH) is the most common cause of lower back pain and sciatica, with an estimated prevalence of 3% to 5%.^[1,2]^ Patients who experience progressive symptoms usually require surgical interventions, when conservative management has failed.^[3,4]^ Conventional open discectomy (OD), open microdiscectomy with a microscope, and endoscopic discectomy techniques (ED) including percutaneous endoscopic lumbar discectomy (PELD), micro-endoscopic discectomy (MED) are principal surgical procedures used to treat LDH.

Conventional OD has become a gold standard procedure in treating LDH. However, OD has been criticized because it requires muscle retraction, bone resection of the lamina and facet joint, dural sac, and nerve retraction, which results in iatrogenic instabilities^[5,6]^ and failed back syndromes.^[7]^ Hence, conventional OD has been gradually replaced by bone-sparing techniques. Minimally invasive techniques, such as ED, involve even smaller incisions with the aid of endoscopic visualization and illumination.^[8]^

Compared with OD, ED procedure is a relatively new technique for treatment of LDH with minimal risk of complications and preserving normal anatomy.^[9]^ It is not unusual to be fascinated by new techniques, but which might lead to a misuse. ED techniques have potential benefits of faster recovery, reduced complications and improved visualization of the anatomy. However, the safety of these techniques has been questioned due to the complexity of C-arm guided orientation, difficulty to find the optimal trajectory for target, more steps of surgical manipulation and small working space, which might make it difficult to avoid the damage to dural and neural structures. It was reported that there were more dural tear, root injury, and recurrence in the ED techniques.^[10–12]^ Furthermore, ED technique requires expensive operating equipment, while accompanied by more x-ray radiation exposure. Current evidence is insufficient to support the better efficacy of ED over OD procedure.^[13]^ Optimal surgical technique to treat LDH remains controversial.

Therefore, we described a small incision discectomy technique (SID) for the treatment of LDH. The aim of this study was to evaluate its safety and efficacy.

## 2 Materials and methods

This study was approved by the Ethics Committee of the Peking Union Medical College Hospital and informed consent was obtained. The data of the consecutive hospitalized patients with LDH treated with SID between June 2007 and February 2012 were collected. SID was performed by the same spine surgeon, who had longer than 10-year experience with the use of OD at the start of study in our spine center. Among these patients, 98 patients were suitable for our study. The demographic characteristics of the patients were recorded. All patients were evaluated before surgery by computed tomography (CT) and magnetic resonance imaging (MRI) to determine the location of the disc herniation and the presence of calcification. Standard anteroposterior and lateral lumbar radiographs were obtained to detect scoliosis, spondylolisthesis, or spinal instability.

### 2.1 Inclusion and exclusion criteria

Inclusion criteria included: single-level symptomatic LDH with a corresponding neural compression on preoperative MRI and CT scans; neurological examination showed motor weakness, sensory changes, or the presence of abnormal reflex; unsuccessful conservative treatment for at least 12 weeks; age of 18 to 60 years at time of surgery. Exclusion criteria were: lateral type of disc herniation; cauda equina syndrome; calcified disc herniation; revision surgery; severe spinal stenosis; spondylolisthesis, or significant lumbar spinal instability; coexisting scoliosis.

### 2.2 Surgical technique of small incision discectomy

**Figure 1:**
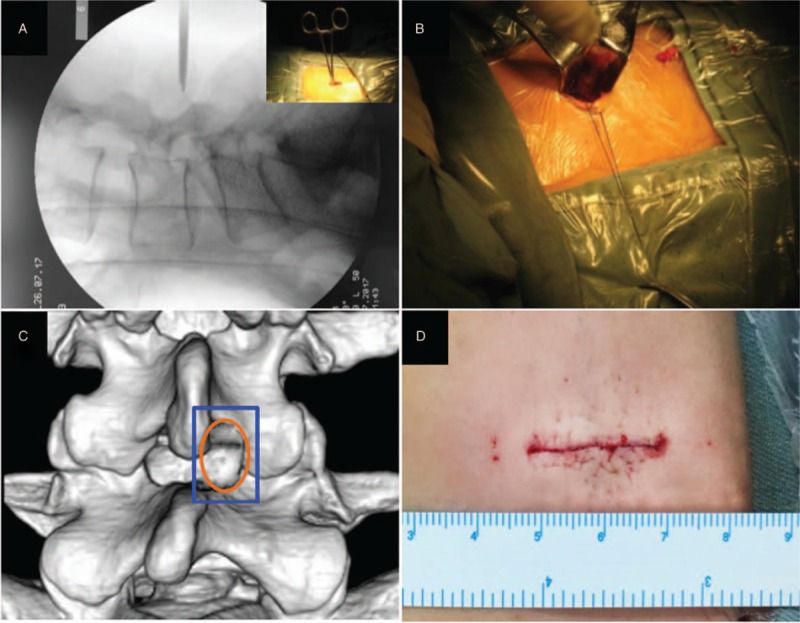
Procedures of small incision discectomy. A: C-arm image to identify the correct vertebral plate gap. B: Two thyroid retractors to expose the operative field. C: The blue circle showed the operative field. A small part of inferior border of the superior lamina and superior border of the inferior lamina were removed with Kerrison rongeurs as shown in the orange circle. D: Placing the drainage tube and suturing the incisions.

Surgery was carried out under general anesthesia. All patients were operated upon with a posterior approach in prone position, with the abdomen free, by the same spine surgeon using a headlight with magnifying glass (2.5 times magnification). The disc space to be operated on was located by palpating the iliac crests and spinous processes. The corresponding level was verified by putting a marker overlying the disc space, and taking a C-arm image. Then a longitudinal skin incision of approximately 2 cm was made in the midline, centering at the level of the disc space. The subcutaneous tissue was dissected with monopolar electrocautery, then the lumbodorsal fascia was incised. Paravertebral muscle was dissected laterally from the spinous processes, lamina, and the medial facet joint only on the symptomatic side in the subperiosteal plane. The inferior border of the superior lamina, medial border of the inferior articular process, and the superior border of the inferior lamina were verified by using a hemostatic forcep. X-ray fluoroscopy was used to identify the correct disc space again (Fig. 1A). The operative field was exposed with 2 thyroid retractors. The contralateral skin was suspended with 7# silk to further expose the field (Fig. 1B). Identifying the ligamentum flavum, then a small part of inferior border of the superior lamina and superior border of the inferior lamina were removed with Kerrison rongeurs (Fig. 1C). The next step was to remove the ligamentum flavum (LF) and gain access to the epidural space, which can be done as follows: the LF was separated bluntly with small dissectors such as nerve root retractors in a longitudinal manner and subsequently removed with Kerrison rongeurs. Epidural fat occasionally obscuring the field should be removed with pituitary rongeurs. Then the compressed dural sac and nerve root were exposed and tracked contralaterally to localize the herniated disc. The nerve root was retracted medially using an atraumatic nerve root retractor to access the ventral epidural space. If the posterior longitudinal ligament was intact, an incision (3–5 mm) was performed and blunt nerve hooks can be used to mobilize free disc fragments that can be then taken with pituitary rongeurs. Only the pathological disc material was removed. The remaining in the disc space was preserved as much as possible. Following discectomy, the disc space was washed with sterile saline to swill out the remaining fragments. After disc removal, the epidural space was explored with careful attention directed to the foramen to ensure that the nerve root had unrestricted passage. Drainage tube was placed in the surgical wound. After hemostasis was achieved, the wound was closed in standardized fashion (Fig. 1D).

### 2.3 Postoperative management

Patients were mobilized on the first postoperative day. Analgesia was prescribed on an as-needed basis. Twenty four hours drainage <50 mL was considered as the standard for the removal of drainage tube. Patients were routinely discharged 2 to 3 days after surgery.

### 2.4 Evaluation

Outcomes included 10-point visual analogue scale pain scores (VAS) for low back pain and leg pain, and 29-point Japanese Orthopedic Association scores (JOA), all of which were obtained preoperatively, postoperative immediately, and subsequently at 1, 3, 6, 12, 24, 36, 48, 60 months at the follow-up after surgery. All patients received the appropriate questionnaire by mail 4 working days prior to each time-point. The clinical outcomes were determined to be excellent, good, fair, or poor according to the MacNab classification^[14]^ (Table 1) at the last follow-up time. The fair and good grades also included that the patients were willing to select the same procedure again for the same problem in the future. The mandatory poor grade was given to patients who had undergone reoperation subsequently at the same level.

**Table 1:**
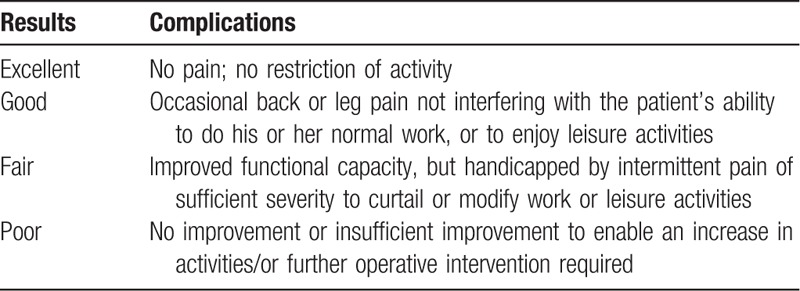
MacNab classification^[10]^.

Additionally, operative time, blood loss, hospital stay, overall hospitalization costs, intraoperative x-ray exposure, reoperation, and complications were reviewed by patient files. Blood loss included intraoperative blood loss plus wound drainage. All complications were registered including iatrogenic nerve damage, dural tear, vascular injuries, surgical site infection, or thrombosis perioperatively, and during the 5-year follow-up period. We also retrospectively collected the overall hospitalization costs data involving 50 patients with LDH treated by PELD conducted by another group in our center. The overall hospitalization cost of SID and PELD was then compared.

### 2.5 Data analysis

All statistical analyzes were performed with SPSS statistical software (version 23.0, SPSS, Chicago, IL). Categorical variables were summarized as the number and proportion, and continuous variables were summarized as the mean and standard deviation. Paired *t* tests were performed to compare pre- and postoperative scores on VAS and JOA. The costs between the groups were compared using independent *t* test. Two-sided values of *P* <.05 were considered statistically significant.

## 3 Results

### 3.1 Patient demographics

Demographic data are summarized in Table 2. Ninety eight patients, 42 women and 56 men, were included in the study. The mean age at the time of operation was 35.7 ± 7.2 years. The follow-up time was 60 ± 2.1 months. The most common level of LDH was L4–5 (55.1%), followed by L5–S1 (41.8%), and L3–4 (3.1%). The location of herniation was central (31.6%) and paramedian (68.4%). Ninety five (96.9%) patients completed the 5-year follow-up.

**Table 2:**
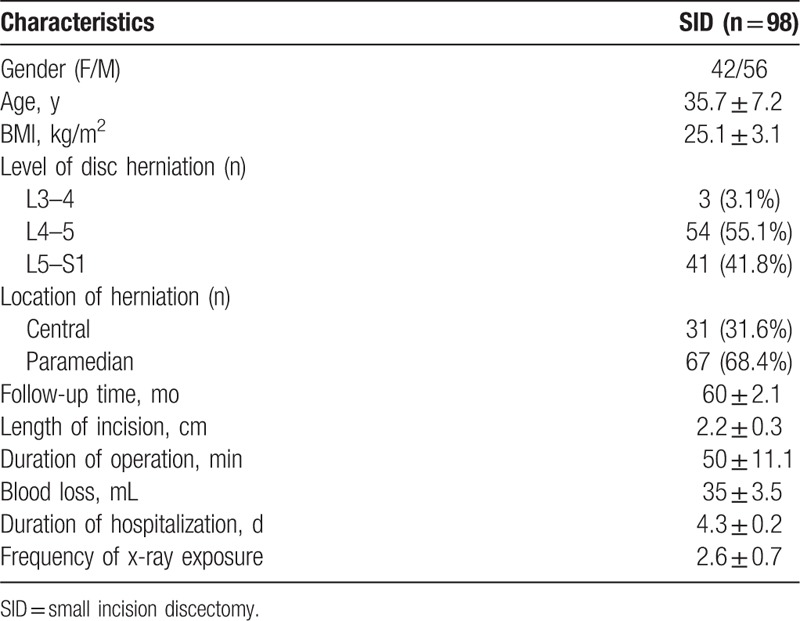
Demographic values.

### 3.2 Outcomes

**Figure 2:**
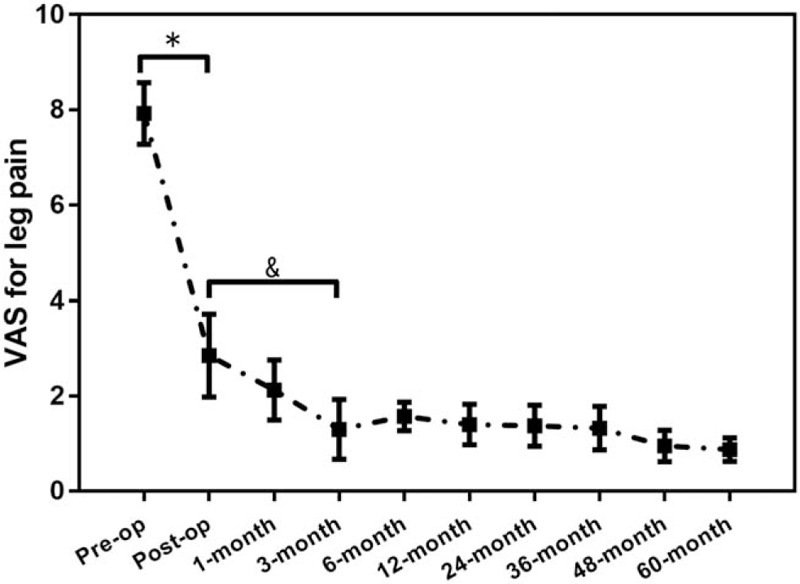
The pre- and postoperative VAS for leg pain. ^∗^*P* < .001, ^&^*P* < .05. VAS = visual analogue scale.

**Figure 3:**
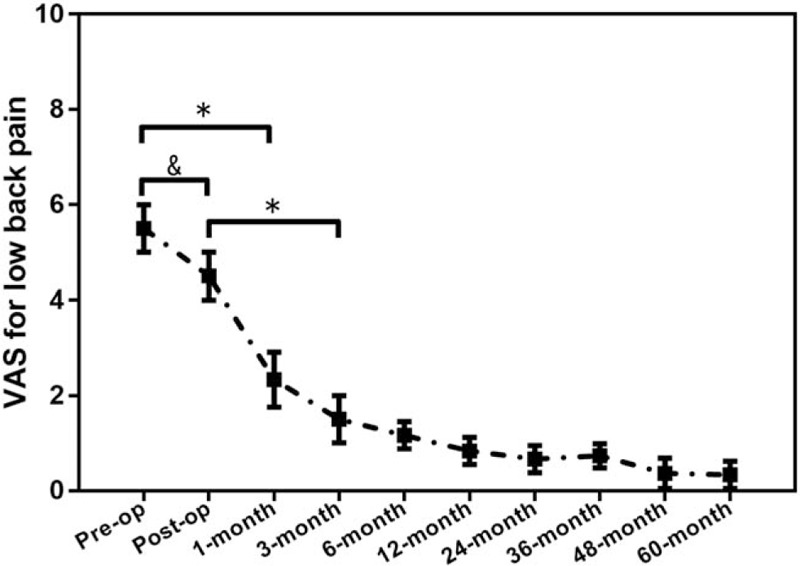
The pre- and postoperative VAS for low back pain. ^∗^*P* < .001, ^&^*P* < .05. VAS = visual analogue scale.

**Figure 4:**
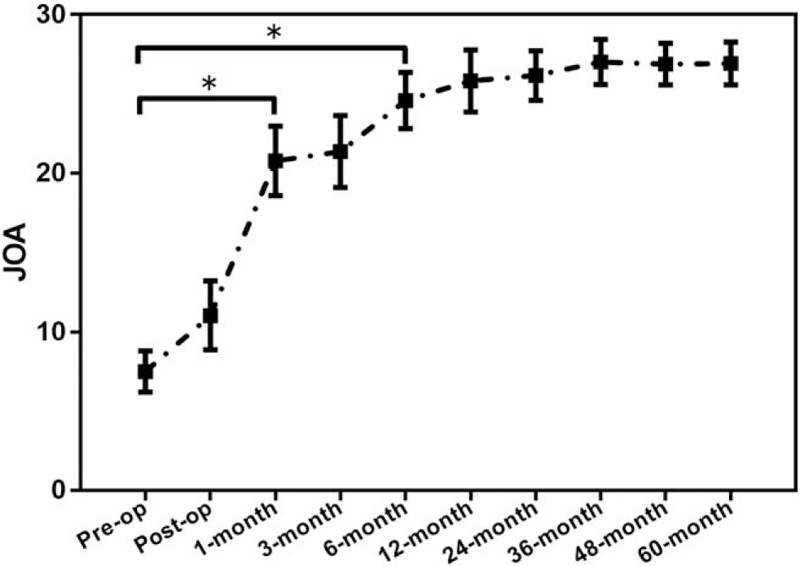
The pre- and postoperative JOA. ^∗^*P* < .001. JOA = Japanese Orthopedic Association.

VAS for leg pain (Fig. 2) improved from 8.2 ± 0.9 preoperatively to 1.0 ± 0.5 points postoperatively (*P* < .001). VAS for low back pain (Fig. 3) improved from 5.0 ± 0.9 preoperatively to 0.5 ± 0.5 points postoperatively (*P* < .001) at the last follow-up time. JOA scores (Fig. 4) improved from 7.0 ± 2.0 points preoperatively before surgery to 27.7 ± 1.0 points postoperatively (*P* < .001) at the last follow-up time. At 5-year follow-up, 93.8% (92/98) of the patients showed excellent or good results, and 3.1% (3/98) fair. Three (3.1%) patients were rated poor because they required subsequent fusion surgery within the 5-year follow-up period.

**Figure 5:**
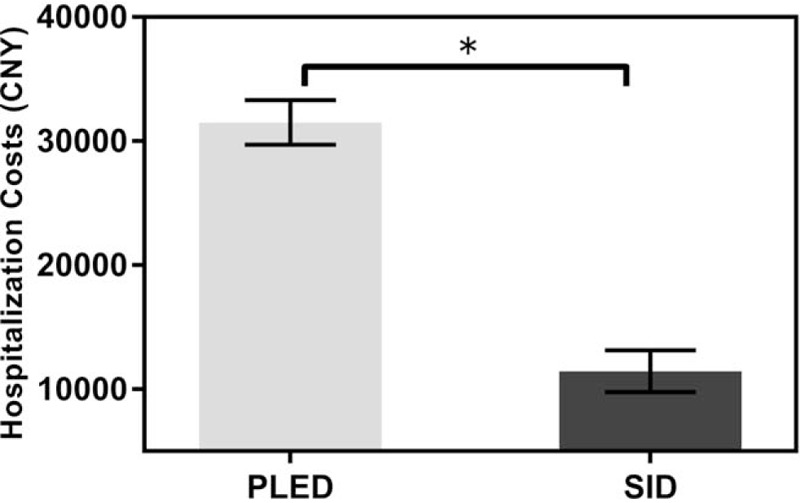
The overall hospitalization costs. ^∗^*P* < .001.

The operation time, length of incision, blood loss, and hospital stay were 50 ± 11.1 minutes, 2.2 ± 0.3 cm, 35 ± 3.5 mL, and 4.3 ± 0.2 days, respectively (Table 2). Moreover, cost analyzes in Fig. 5 showed that overall hospitalization costs were significantly higher in PELD group (31,578 ± 2060 CNY), >2 times as much as SID group (14,749 ± 3217 CNY, *P* < .0001). Furthermore, frequency of intraoperative x-ray exposure was only 2.6 ± 0.7 (Table 2), whose single exposure time was 2 seconds, and the cumulative duration of x-ray exposure was only a few seconds. The reoperation rate was 3.2% (3/95), all of which underwent fusion surgery subsequently: epidural scar adhesion (n = 1), recurrent herniation (n = 2). No cases of iatrogenic nerve damage, dural tear, vascular injuries, surgical site infection, or thrombosis were observed.

## 4 Discussion

In our study, SID was achieved through an interlaminar approach without special expensive equipment. We compared pre- and postoperative scores, demonstrating that there was constant and significant improvement in VAS for leg pain and low back pain, and JOA scores throughout the whole follow-up period. Moreover, 93.6% (89/95) of the patients showed excellent or good results, 3.2% (3/95) fair, and 3.2% (3/95) poor. Outcomes at the 5-year follow-up appear to be satisfactory, indicating that SID leads to substantial clinical outcomes for treatment of LDH.

The potential problems of ED techniques include longer operating time that reflects the learning curve inherited to this video-endoscopic technique and the complex hand-eye coordination required. Chen et al^[15]^ and Choi et al^[16]^ have reported that the operation time of PELD are 79 ± 31 and 67 ± 12 minutes, respectively. And those of MED are 84 ± 36 and 98 ± 26 minutes, reported by Garg et al^[17]^ and Hussein et al.^[18]^ In our study, the operation time was 50 ± 11.1 minutes, which was less than ED procedures (PELD and MED) of the previous study. Furthermore, the length of incision in our study was 2.2 ± 0.3 cm, which was even comparable with that in ED procedures (ranged from 0.8 to 2.5 cm).^[19–22]^ In addition, according to the study of Pan et al,^[23]^ the blood loss of PELD is 8.4 ± 2.9 mL. And, on the basis of results from Garg et al,^[17]^ Huang et al,^[24]^ and Hussein et al,^[18]^ the blood loss of MED is 41 ± 12, 87 ± 69, and 41 ± 13 mL, respectively. In our study, it was 35 ± 3.5 mL, which was more than PELD and less than MED. However, the differences were not large, and may not have been clinically significant. Moreover, this study also showed that the hospital stay (4.3 ± 0.2 days) was longer than that of ED reported by Phan et al^[25]^ (ranged from 1 to 2 days). This may be due to that there is no mature rehabilitation hospital support system in China.

In our study, the overall hospitalization costs were significantly higher in PELD technique, >2 times as much as SID in our center (*P* < .0001), indicating that SID technique may be more cost-effective. Besides, there are more steps of manipulation during PELD procedure, which lead to considerable exposure of x-ray. The entire x-ray exposure time of PELD is 78 ± 48 seconds according to Sencer research.^[26]^ In our study, the frequency of intraoperative x-ray exposure were 2.6 ± 0.7 times, and the cumulative duration was just a few seconds, which was significantly shorter than the entire x-ray exposure time of PELD reported by Sencer.^[26]^

ED such as PLED and MED in spinal surgery has a very steep learning curve and could be hazardous to the patients at the early periods of learning curve.^[27,28]^ The perceived steep learning curve has discouraged many clinicians. It was reported that, at the beginning of the learning curve, the poor perception of depth with endoscopic surgery is linked to higher incidence of iatrogenic dural and root injuries,^[11,29]^ while the restricted working space by tubular retractor might justify lower chance of identifying and removing free fragments within the disc space, ultimately leading to higher recurrences.^[21]^ That's why we use 2 thyroid retractors rather than tubular retractor to expose the working field, which is very convenient to adjust independently at any time during surgery. In a large single-center retrospective review of 10,228 cases, the scholars found a short-term recurrence rate of 4.3% and reoperation rate of 4.2% to 11.0% in the PELD group.^[30]^ Furthermore, other studies report similar results.^[31–34]^ Nevertheless, the reoperation and recurrence rate in our study were 3.2% and 2.1%, respectively.

The complications in the aforementioned study include iatrogenic nerve damage, dural tear, vascular injuries, or surgical site infection,^[19,25,35,36]^ while none of which were observed in our study. The most important reason for this difference could be that SID technique is quite friendly to master for beginners, which is suitable for young surgeons. Like with most new technologies, the using of SID may be also associated with a learning curve. However, for surgeons who are proficient in conventional OD, SID technique is easier to grasp because of similarities in the anatomic orientation. On the contrary, the PELD technique may be more demanding. Besides, the implementation of SID requires no expensive equipment, which is especially applicable to developing countries, for instance China. It's worth pointing out that our SID technique is less suitable for lateral type of disc herniation, due to its own limitations of interlaminar approach.

The strength of our study in the homogeneity of the patients treated for symptomatic LDH by the same surgeon. Moreover, the follow-up period was relatively long and follow-up rate was also remarkable. In addition, this study has several limitations. Firstly, there was no investigation on quality of life scores. Secondly, due to the nature of observational study, it lacked a control group. The comparative data came from previous literature, so there may be differences in national conditions. Therefore, prospective randomized controlled studies are needed to further evaluate the possible advantages of SID over other techniques.

## 5 Conclusion

SID leads to substantial clinical outcomes at mid-term follow-up and appears to be a safe, cost-effective technique for symptomatic LDH, and has lower x-rays exposure time when compared with literature of PELD.

## Author contributions

**Data curation:** Zhinan Ren.

**Formal analysis:** Zhinan Ren, Zheng Li.

**Methodology:** Zheng Li.

**Project administration:** Shugang Li.

**Software:** Derong Xu, Xin Chen.

**Supervision:** Shugang Li.

**Writing – original draft:** Zhinan Ren.
